# Antispasmodic and bronchorelaxant activities of *Salsola imbricata* are mediated through dual Ca^+2^ antagonistic and β-adrenergic agonistic effects

**DOI:** 10.1080/13880209.2017.1291691

**Published:** 2017-02-17

**Authors:** Naveed Aslam, Khalid Hussain Janbaz

**Affiliations:** a Department of Pharmacy, Bahauddin Zakariya University, Multan, Pakistan;; b Akson College of Pharmacy, MUST, Mirpur, Azad Kashmir, Pakistan

**Keywords:** *Salsola baryosma*, *Salsola foetida*, isolated tissue, jejunum, trachea

## Abstract

**Context:***Salsola imbricata* Forssk. (Chenopodiaceae) has folkloric repute for the treatment of various gastrointestinal and respiratory ailments.

**Objective:** The present study investigates spasmolytic and bronchorelaxant effects of *S. imbricata.*

**Materials and methods:** The crude aqueous-ethanol extract of the aerial parts of *S. imbricata* and its fractions, in cumulative concentrations (0.01–10 mg/mL), were tested on contractions of isolated rabbit jejunum and tracheal preparations. Furthermore, concentration response curves (CRCs) of Ca^+2^ and carbachol were constructed in the absence and presence of the extract. Standard organ bath methods were used.

**Results:** The crude extract relaxed spontaneous, K^+ ^(80 mM) and carbachol (1 μM)-induced contractions in jejunum preparations with respective EC_50_ values of 0.40 (0.35–0.46), 0.69 (0.60–0.79) and 0.66 (0.57–0.75) mg/mL. It shifted Ca^+2^ CRCs rightward in nonparallel manner. In isolated tracheal preparations, the crude extract caused relaxation of K^+ ^(80 mM) and carbachol (1 μM)-induced contractions with EC_50_ values of 0.86 (0.75–0.98) and 0.74 (0.66–0.84) mg/mL, respectively. It displaced carbachol CRCs rightward with suppression of maximal response. In both tissues, pretreatment with propranolol (1 μM) caused rightward shift in inhibitory CRCs of the extract against carbachol-induced contractions. The ethyl acetate fraction was found more potent in relaxing smooth muscle contractions than the parent extract and its aqueous fraction.

**Discussion and conclusion:** The results suggest that the spasmolytic and bronchorelaxant activities of *S. imbricata* are related to Ca^+2^ antagonistic and β-adrenergic agonistic effects, thus justifying some of the traditional uses of the plant.

## Introduction

*Salsola imbricata* Forssk. (Chenopodiaceae) is a shrub that grows on saline and sandy places with distribution from west and southwest Saharan countries throughout hot desert belt to tropical east Africa, south Iran, Pakistan, south and east Afghanistan and northwest India. The plant is also known by synonyms *Chenopodium baryosmum* Schult., *Salsola foetida* Del., *Caroxylon foetidum* Moquin. and *Salsola baryosma* Schult. (Boulous 1991). Local names include Haram, Lani, and Lana. In folk medicine, it is used in treatments of indigestion, diarrhoea, dysentery, cold, asthma (Ahmed et al. 2014; Malik et al. 2015) and sinus congestion (Handa et al. 2006). The plant is also reported as a vermifuge (Farooq et al. 2008), diuretic (Phondani et al. 2016) and female contraceptive (Ahmed et al. 2014).

Phytochemical analysis of *Salsola imbricata* reported the presence of alkaloids, anthraquinones, tannins, saponins and flavonoids in various extracts of the plant (Mahdavi & Mir 2006; Hamed et al. 2011; Munir et al. 2014). Coumarins including scopoletin, bergaptol, bergaptol glycosides, daphnoretin, daphnorin and chrysoeriol glucoside have been isolated from methanol extract of the plant (Ahmed et al. 2006b). Quercetin and coumaric acid have been identified as major phenolics of hydrolyzed ethanol extract of the plant (Shehab & Abu-Gharbieh 2014). This plant from Cholistan desert is reported to contain about 1.23% sodium, 0.42% potassium and 0.23% calcium on dry weight basis (Abdullah et al. 2013).

Pharmacological investigations revealed that the plant has antioxidant (Ahmed et al. 2006a), male contraceptive (Shehab & Abu-Gharbieh 2014), antibacterial (Kaur & Bains 2012), butyrylcholinesterase (Ahmed et al. 2007), and tyrosinase inhibitory (Khan et al. 2003) activities. An ethanol extract of the plant was found to have spasmolytic action in rabbit jejunum (Ahmed et al. 2006a). However, mechanisms of spasmolytic action were not investigated. No scientific report is available regarding respiratory tract effects of the plant. The purpose of present study was to investigate therapeutic potential and possible mechanism(s) of action which may explain the traditional uses of *S. imbricata* in gastrointestinal and respiratory disorders involving spasmodic conditions.

## Materials and methods

### Plant material

Aerial parts of *Salsola imbricata* were collected in the month of May 2014 from Cholistan desert, district Bahawalpur, Pakistan. The plant material was identified by taxonomist Prof. Dr. Altaf Ahmed Dasti, Department of Botany, Institute of Pure and Applied Biology, Bahauddin Zakariya University, Multan and a voucher number Fl.P.225-9 was assigned to sample of the plant material. The plant material was rendered free from extraneous matter and dried in shade for 14 d.

### Preparation of extract and fractions

The dried plant material was converted into coarse powder with help of an electric grinder and soaked in aqueous-ethanol (30:70 v/v) at room temperature with occasional stirrings for 3 d followed by filtration through muslin cloth and then through Whatman® grade 1 filter paper. The procedure of soaking and filtration was repeated with the residue using fresh solvent for two more times. All the three filtrates were combined for evaporation under reduced pressure at temperature not exceeding 45 °C to yield a thick semisolid mass of greenish brown colour; i.e., the crude extract of *Salsola imbricata* (Si.Cr). A part of crude extract was subjected to fractionation by suspending about 50 g of the crude extract in 50 mL water in a separating funnel and same volume of petroleum ether added, shaken vigorously with periodical removal of air from the funnel and mixture was allowed to separate into two layers for about 20–30 min. The organic layer was removed and extraction with the same solvent was repeated for two more times. All the same fractions were combined and evaporated on rotary evaporator to yield petroleum ether fraction (Si.Pet). The same procedure was repeated with ethyl acetate for respective fraction (Si.EtAc). The remaining aqueous residue was also dried on rotary evaporator to yield aqueous fraction (Si.Aq). Percentage yield of Si.Cr, Si.Pet, Si.EtAc and Si.Aq were found 8.9, 0.2, 0.3 and 8.3% of the dried plant material, respectively. The extract and fractions were stored at −4 °C until used. Petroleum ether fraction was not studied due to limited availability and poor solubility. Si.Cr and Si.Aq were dissolved in distilled water and Si.EtAc was dissolved in 30% DMSO. Solutions were freshly prepared on every day of the experiments.

### Animals

Locally available breed of rabbits of either sex, weighing between 1.5 and 2 kg, purchased from local market and maintained at the animal house of the Department of Pharmacy, Bahauddin Zakariya University, Multan, Pakistan at temperature of 25 ± 2 °C and exposed to 12 h light–dark cycles were used in experiments. The animals had free access to water and a standard diet. Guide for the care and use of laboratory animals issued by Institute of Laboratory Animal Research, Commission on Life Sciences, NRC was followed and the study was approved by the Ethical Committee of Bahauddin Zakariya University, Multan, Pakistan.

### Chemicals and reagents

Atropine sulfate, acetylcholine chloride, carbachol, verapamil hydrochloride, potassium chloride, magnesium chloride and ethylenediaminetetraacetic acid (EDTA) were purchased from Sigma Chemicals Co. St. Louis, MO. Potassium-di-hydrogen phosphate, glucose, calcium chloride, magnesium sulfate, sodium bicarbonate, sodium-di-hydrogen phosphate and ethanol were purchased from Merck, Darmstadt, Germany. Sodium chloride was purchased from BDH Laboratory Supplies, Poole, UK. All chemicals used were of the analytical grade and dissolved in distilled water.

### Studies on isolated jejunum preparations

Rabbits were sacrificed by cervical dislocation, abdomens opened, jejunums isolated, cleaned off from the connective tissues and cut into pieces of about 2 cm. Each piece was mounted in a 15 mL organ batch (Radnoti Tissue Organ Bath System, AD Instruments, Australia) containing Tyrode’s solution (having composition of KCl 2.68, NaCl 136.9, MgCl_2_ 1.05, NaHCO_3_ 11.90, NaH_2_PO_4_ 0.42, CaCl_2_ 1.8, and glucose 5.55 mM), maintained at 37 °C and continuously bubbled with carbogen (95% O_2_ and 5% CO_2_). Spontaneous contractions were recorded under preload of 1 g tension by using force displacement transducer connected to a PowerLab data acquisition system (AD Instruments, Australia). The tissue was allowed to equilibrate for about 30 min with fluid changing at interval of every 10 min before addition of any drug. After equilibration, the tissue was repeatedly treated with sub-maximal doses of acetylcholine (0.3 μM) with 3 min intervals between doses to stabilize the preparation. The test material was then added on the spontaneous contracting jejunum preparations in cumulative doses. The inhibitory effects of test materials were calculated as percent change in spontaneous contractions of jejunum obtained immediately before addition of the test substances. In another set of experiments, K^+ ^(80 mM), as KCl, was applied on isolated tissue as spasmogenic agent and after achieving sustained contraction, the test substance was then added to the organ bath in cumulative doses. The substance that relaxes the contractions induced by K^+ ^(80 mM) is considered as Ca^+^^2^ channel blocker. Ca^+^^2^ antagonist property of the test substance was confirmed by stabilizing the tissue in normal Tyrode’s solution, which was then replaced with Ca^+^^2^ free Tyrode’s solution containing EDTA (0.1 mM) for 30 min to remove Ca^+^^2^ from the tissues. This solution was further replaced with K^+ ^rich and Ca^+^^2^ free Tyrode’s solution having composition of KCl 50, NaCl 91.04, MgCl_2_ 1.05, NaHCO_3_ 11.90, NaH_2_PO_4_ 0.42, glucose 5.55, and EDTA 0.1 mM. Following an incubation period of 30 min, control concentration response curves (CRCs) of CaCl_2_ were obtained. When the control CaCl_2_ CRCs were found super-imposable (usually after two cycles), the tissue was pretreated with the crude extract for 60 min and CRCs of CaCl_2_ were reconstructed in the presence of different concentrations of the extract (Janbaz et al. 2015).

To study involvement of additional mechanisms in spasmolytic action of the extract, the test substance was tested on carbachol (1 μM)-induced contractions. Carbachol, a cholinergic agonist causes smooth muscle contraction through activation of cholinergic muscarinic receptors. After achieving sustained contraction (usually after 10–15 min), the test material was then added in cumulative doses to obtain concentration dependant inhibitory response. In another set of experiments, tissue was incubated with propranolol (1 μM) for 15 min before induction of contraction with carbachol and the extract was then added in cumulative doses. The obtained concentration-dependant inhibitory responses were compared to find involvement of β-adrenergic activity in spasmolytic action of the extract (Bashir et al. 2011; Daniel et al. 2001).

### Studies on isolated tracheal preparations

The trachea of euthanized rabbit was dissected out, cut into rings containing about two cartilages and opened by a longitudinal cut on ventral side opposite to the smooth muscle layer, forming a tracheal strip with a central part of smooth muscle sandwiched between cartilaginous portions on edges. The preparation was suspended with help of cotton thread in a 15 mL tissue organ batch containing Kreb’s Henseleit solution (having composition of NaCl 118.2, NaHCO_3_ 25.0, CaCl_2_ 2.5, KCl 4.7, KH_2_PO_4_ 1.3, MgSO_4_ 1.2 and glucose 11.7 mM), continuously bubbled with carbogen and maintained at 37 °C. The tissue was equilibrated for 45 min at 1 g tension before exposure of the test material, during which bathing solution was changed after every 15 min. The change in isometric contraction was recorded via force displacement transducer connected to PowerLab data acquisition system (AD Instruments, Australia). After equilibration, the preparation was stabilized by repeated administrations of carbachol (0.3 μM) before addition of the test material. The relaxant effect of the test material was assessed on carbachol (1 μM) or KCl (80 mM)-induced contractions in isolated tracheal preparations as the cumulative addition of the test material to the isolated tissue bath may relax the isolated tracheal preparations (Janbaz et al. 2015). In some preparations, cumulative CRCs to carbachol were constructed using increasing concentrations of the drug. When a 3-fold increase in concentration produced no further increment in response, the tissue was washed to reestablish the baseline tension. Carbachol CRCs were then repeated in presence of increasing concentrations of the test material. In another set of experiments, propranolol (1 μM) was added to tissue bath 15 min before induction of carbachol (1 μM)-induced contraction and then relaxant effect was assessed by cumulative addition of the test material (Bashir et al. 2011).

### Statistical analysis

The data is expressed as mean ± standard error of mean (SEM) and median effective concentrations (EC_50_) with 95% confidence interval (CI). GraphPad Prism Software (GraphPad, San Diego, CA) was used to analyze data and construct graphs of CRCs by applying nonlinear regression curve fit. Two-way ANOVA followed by Bonferroni’s post-test correction was used to compare CRCs with control and *p* value <0.05 was considered significant.

## Results

### Effects of crude extract on rabbit jejunum

**Figure 1.**
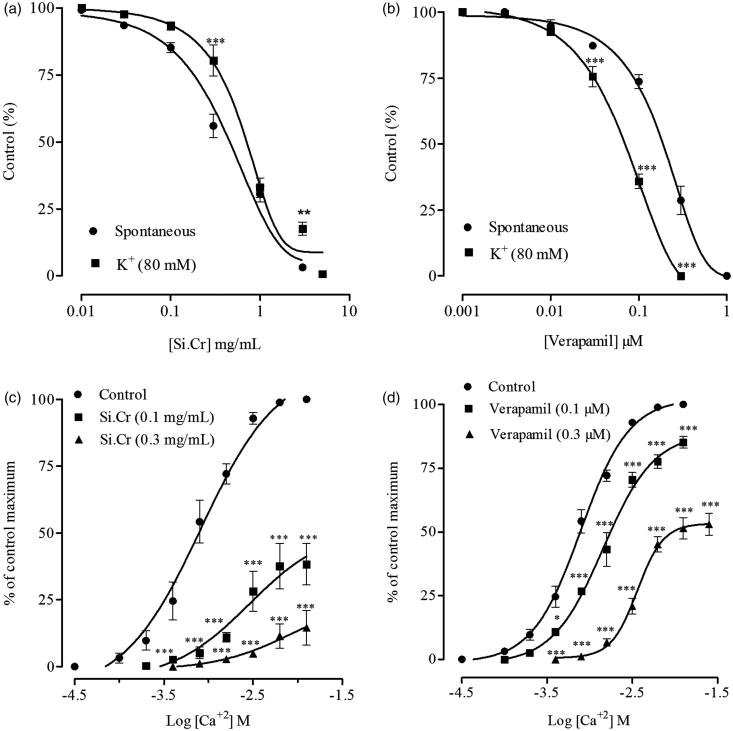
Inhibitory effects of (a) crude extract of *Salsola imbricata* (Si.Cr) and (b) verapamil on spontaneous and K^+^-induced contractions in rabbit jejunum preparations. Concentration response curves of Ca ^+^ ^2^ in the absence and presence of (c) Si.Cr and (d) verapamil constructed in Ca ^+^ ^2^-free and K^+^-rich Tyrode’s solution in rabbit jejunum preparations. Values are mean ± SEM of 5 determinations. *p < 0.05, **p < 0.01, ***p < 0.001 compared to the corresponding concentrations values in spontaneous or control contractions.

**Figure 2.**
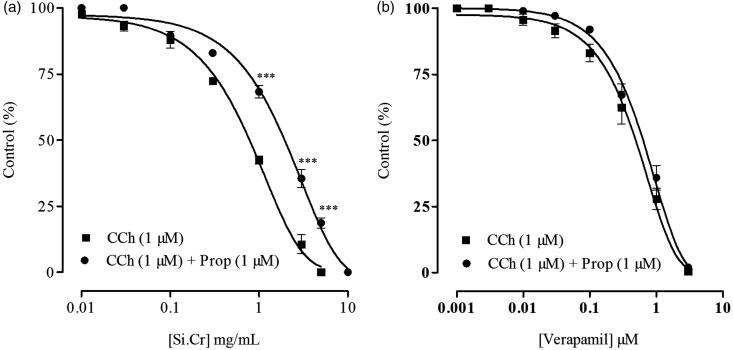
Inhibitory effects of (a) crude extract of *Salsola imbricata* (Si.Cr) and (b) verapamil on carbachol (CCh)-induced contractions in rabbit isolated jejunum preparations in presence and absence of propranolol (Prop). Values are mean ± SEM of 3–5 determinations. ***p < 0.001 compared to the corresponding concentrations values in CCh-induced contractions.

Si.Cr produced relaxant effect on spontaneous, K^+ ^(80 mM) and carbachol (1 μM)-induced contractions in rabbit jejunum preparations (Figures 1(a) and 2(a)) with respective EC_50_ values of 0.40 (0.35–0.46, *n* = 5), 0.69 (0.60–0.79, *n* = 5) and 0.66 mg/mL (0.57–0.75, *n* = 5). Varapamil also relaxed spontaneous, K^+ ^(80 mM) and carbachol (1 μM)-induced contractions with respective EC_50_ values of 0.17 (0.14–0.19, 95% CI, *n* = 5), 0.06 (0.05–0.07, *n* = 5) and 0.42 μM (0.33–0.54, *n* = 5) as shown in Figures 1(b) and 2(b). Pretreatment of tissue with Si.Cr shifted the Ca^+^^2^ CRCs rightwards with suppression of maximum response at 0.1 (*p* < 0.001) and 0.3 mg/mL (*p* < 0.001) as seen in Figure 1(c). Similarly, verapamil at concentrations of 0.1 and 0.3 μM produced a non-parallel shift in Ca^+^^2^ CRCs with suppression of maximum response (*p* < 0.001) as shown in Figure 1(d).

The relaxant effect of Si.Cr on carbachol-induced contractions was decreased significantly (*p* < 0.001) in presence of propranolol (1 μM) with increase in EC_50_ values from 0.66 (0.57–0.75, 95% CI, *n* = 5) to 1.61 mg/mL (1.38–1.87, 95% CI, *n* = 3), but that of verapamil remained unaffected in presence of propranolol (Figure 2).

### Effect of crude extract on rabbit trachea

**Figure 3.**
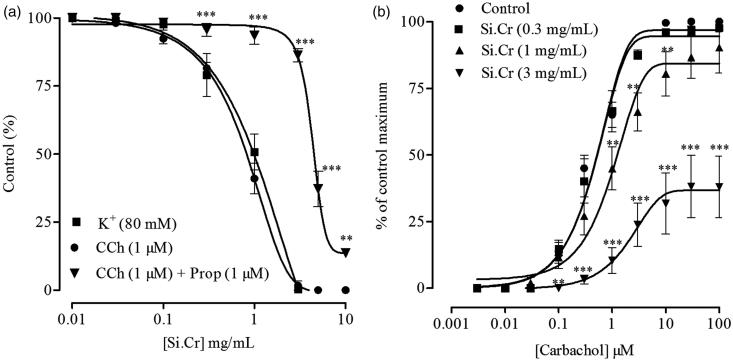
(a) Inhibitory effects of crude extract of *Salsola imbricata* (Si.Cr) on rabbit isolated trachea preparations pre-contracted with K^+^, carbachol (CCh) and CCh plus propranolol (Prop). (b) Concentration response curves of CCh in the absence and presence of Si.Cr in rabbit tracheal preparations. Values are mean ± SEM of 5 determinations. **p < 0.01, ***p < 0.001 compared to the corresponding concentrations values in CCh-induced or control contractions.

When tested on isolated rabbit trachea, Si.Cr inhibited K^+ ^(80 mM) and carbachol (1 μM)-induced contractions with respective EC_50_ values of 0.86 mg/mL (0.75–0.98, 95% CI, *n* = 5) and 0.74 mg/mL (0.66–0.84, 95% CI, *n* = 5), as shown in Figure 3(a). Pretreatment with propranolol (1 μM) caused a rightward shift in the inhibitory CRCs of Si.Cr constructed against carbachol (1 μM)-induced contraction (Figure 3(a)) with resultant EC_50_ value of 4.47 mg/mL (4.14–4.84, 95% CI, *n* = 5). Si.Cr shifted the carbachol CRCs rightwards with suppression of maximum response at concentrations of 1 (*p* < 0.01) and 3 mg/mL (*p* < 0.001), as shown in Figure 3(b).

### Effect of fractions on rabbit jejunum and trachea

**Figure 4.**
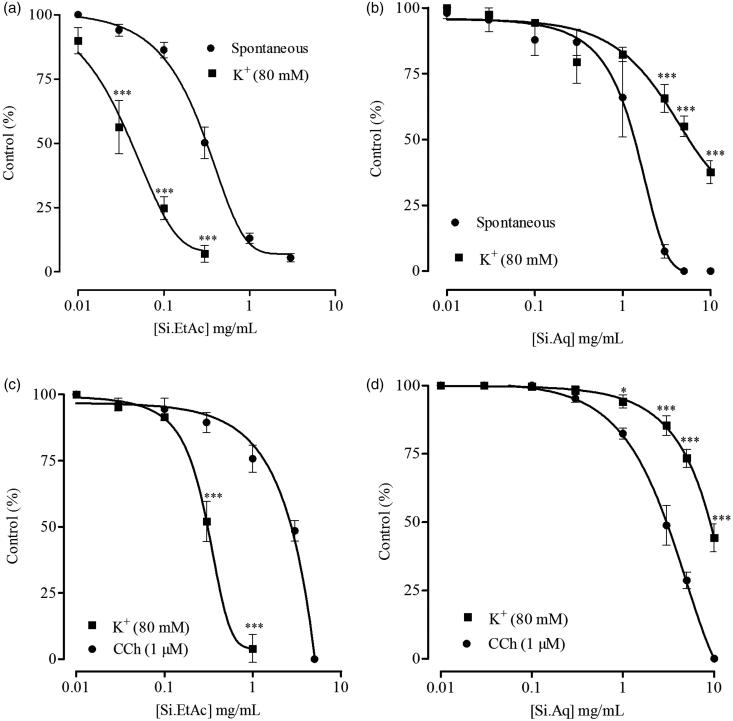
Effects of (a) ethyl acetate (Si.EtAc) and (b) aqueous (Si.Aq) fractions of *S. imbricata* extract on spontaneous and K^+^-induced contractions in rabbit isolated jejunum preparations. Effects of (c) Si.EtAc and (d) Si.Aq on K^+ ^and carbachol (CCh)-induced contractions in rabbit isolated tracheal preparations. Values are mean ± SEM of 4–5 determinations. *p < 0.05, ***p < 0.001 compared to the corresponding concentrations values in spontaneous or CCh-induced contractions.

When tested on isolated rabbit jejunum, Si.EtAc produced relaxations of spontaneous and K^+ ^(80 mM)-induced contractions with EC_50_ values of 0.30 mg/mL (0.27–0.35, 95% CI, *n* = 5) and 0.04 mg/mL (0.3–0.05, 95% CI, *n* = 4), respectively (Figure 4(a)). Si.Aq also produced a spasmolytic effect on spontaneous and K^+ ^(80 mM)-induced contraction in jejunum preparations at dose range of 0.01-10.0 mg/mL with EC_50_ values of 1.25 mg/mL (0.93–1.68, 95% CI, *n* = 5) and 6.24 mg/mL (4.41–8.82, 95% CI, *n* = 5), respectively (Figure 4(b)). In rabbit trachea, Si.EtAc relaxed K^+ ^(80 mM) and carbachol (1 μM)-induced contractions with EC_50_ values of 0.31 mg/mL (0.28–0.33, 95% CI, *n* = 4) and 2.01 mg/mL (1.51–2.66, 95% CI, *n* = 5), respectively (Figure 4(c)). While Si.Aq, when tested at dose range of 0.01–10 mg/mL, caused partial relaxation of K^+ ^(80 mM)-induced contractions with EC_50_ value of 8.87 mg/mL (8.29–9.49, 95% CI, *n* = 5) and complete relaxation of carbachol (1 μM)-induced contractions with EC_50_ value of 2.76 mg/mL (2.45–3.11, 95% CI, *n* = 4) as shown in Figure 4(d).

## Discussion

Keeping in view of various reports on ethnic uses of *Salsola imbricata* in gastrointestinal and respiratory ailments, the present study was undertaken to justify the traditional uses of the plant. Our study evaluates spasmolytic action and mechanisms of action of the aqueous-ethanol extract of the aerial parts of the plant and its fractions on rabbit jejunum and tracheal smooth muscles.

The crude extract (Si.Cr) and its factions (Si.EtAc and Si.Aq) caused dose dependant inhibition of spontaneous contractions in rabbit jejunum preparations, thus demonstrated spasmolytic action. Jejunum shows rhythmic contractile activity, which is regulated by periodic depolarization and repolarization of smooth muscle cells (Huizinga, et al. 2000) and contractions are medicated during depolarization phase through opening of voltage dependant Ca^+^^2^ channels resulting in increase in cytosolic free Ca^+^^2^ (Bolton 1979). The free Ca^+^^2^ on binding to calmodulin causes activation of myosin light chain kinase, which in turn activates contractile proteins. Increase in intracellular free Ca^+^^2^ may be due to influx via ligand or/and voltage gated Ca^+^^2^ channels or to release from intracellular stores from sarcoplasmic reticulum (Karaki et al. 1997). It has been previously observed that spasmolytic effect of plant extracts is usually mediated through voltage dependant Ca^+^^2^ channel blocking activity (Bashir et al. 2011; Janbaz et al. 2015). The involvement of Ca^+^^2^ channel blocking activity in the spasmolytic action is assessed by ability of test substance to relax K^+ ^(80 mM)-induced contractions in smooth muscle preparations (Janbaz et al. 2014). K^+ ^(80 mM) depolarizes the smooth muscle cell membrane, resulting in activation of voltage dependent Ca^+^^2^ channels that lead to influx of extracellular Ca^+^^2^, increase in cytosolic free Ca^+^^2^ and a substance causing inhibition of K^+ ^(80 mM)-induced contractions is considered as a blocker of Ca^+^^2^ influx (Karaki et al. 1997). In our study, Si.Cr and Si.EtAc relaxed K^+ ^(80 mM)-induced contractions, similar to that of verapamil, a standard Ca^+^^2^ antagonist (Katzung & Chatterjee 2009). However, studies demonstrated that K^+ ^(20-120 mM), in addition to increasing intracellular free Ca^+^^2^ by opening voltage dependent L-type Ca^+^^2^ channels, also promote smooth muscle contractions by various other mechanisms, such as release of intracellular Ca^+^^2^ in rabbit intestine, inhibition of myosin light chain phosphatase and release of chemical transmitters from adjacent cells (Grasa et al. 2004; Ratz et al. 2005). Therefore, it should be noted that other mechanisms may also be involved in the relaxant effect of the extract on K^+ ^(80 mM)-induced contractions. However, presence of non-competitive Ca^+^^2^ antagonist activity was confirmed when pretreatment of the tissue with Si.Cr shifted the Ca^+^^2^ CRCs to rightward with suppression of maximum effect (Daniel et al. 2001). Ca^+^^2^ channel blockers constitute an important therapeutic class of drugs that are capable of smooth muscle relaxation by inhibition of Ca^+^^2^ entry through voltage-dependant Ca^+^^2^ channels (Katzung & Chatterjee 2009).

The extract and fractions were also studied on carbachol contracted jejunum preparations. Carbachol, a muscarinic receptor agonist, produce sustained contractions in smooth muscle preparations by increasing cytosolic free Ca^+^^2^ through its release from internal stores as well as influx via voltage dependant Ca^+^^2^ channels (Grasa et al. 2004). Si.Cr relaxed carbachol contracted jejunum preparations with similar potency that relaxed the K^+ ^contracted preparation. Previous studies reported that pure Ca^+^^2^ channel blockers are more effective against K^+^-induced contractions and pure anticholinergics are not effective in relaxing K^+^-induced contractions (Gilani et al. 2008). β-Adrenergic agonists are also reported to relax spontaneous, high K^+ ^and carbachol-induced contractions in smooth muscles (Bowman & Hall 1970; Roberts et al. 1999). Therefore, in addition to Ca^+^^2^ channel blocking activity, Si.Cr was studied for β-adrenergic effects in jejunum preparations. Inhibitory CRC of Si.Cr was shifted to right in presence of β-adrenergic blocking drug, propranolol, suggesting presence of β-adrenergic activity in the extract (Daniel et al. 2001; Chaudhary et al. 2012). β-Adrenoceptors via stimulatory G-protein are linked to adenylate cyclase, whose activation results generation of cAMP, which in turn inhibit myosin light chain kinase and voltage dependant Ca^+^^2^ channels resulting in smooth muscle relaxation (Wikberg 1977). β-Adrenoceptor agonist drugs are reported to inhibit gut motility, decrease oesophageal peristaltic pressure, delay orocecal transit and inhibit sigmoid motility and, therefore, are useful as antispasmodic drugs (Ponti et al. 1996).

As *Salsola imbricata* is traditionally being used in asthma, cough and congestion, the bronchodilator potential of the extract and fractions was explored by testing against carbachol (1 μM) and K^+ ^(80 mM)-induced contractions in isolated rabbit tracheal preparations (Chaudhary et al. 2012; Janbaz et al. 2014). Si.Cr relaxed K^+ ^and carbachol-induced contractions completely at similar concentrations, whereas, verapamil was more potent against K^+^-induced contraction, a typical characteristic of a pure Ca^+^^2^ channel blockers (Gilani et al. 2008). Therefore, in addition to Ca^+^^2^ antagonistic activity, some other mechanisms may also be involved in tracheal relaxant effect of Si.Cr. Anticholinergic drugs are also useful in treatment of bronchospastic conditions. To study possibility of cholinergic receptor antagonistic property of Si.Cr, CRCs of carbachol were constructed in presence and absence of the extract. The extract displaced the carbachol CRCs to rightward in a non-parallel fashion with the suppression of the maximum response, suggesting a non-competitive or non-specific anticholinergic action (Kenakin 2009). Adrenergic drugs with β_2_-agonist activity are commonly used as bronchodilator agent and to see whether Si.Cr has β_2_-agonistic activity in tracheal muscles, inhibitory CRCs of Si.Cr on carbachol-induced contractions was tested in the presence of propranolol (1 μM), a nonspecific β-adrenergic receptor agonist. In the presence of propranolol, CRC of Si.Cr on carbachol-induced contraction was shifted to right, indicating involvement of β_2_-adrenergic mechanism in bronchorelaxant activity of the extract (Chaudhary et al. 2012).

Study on fractions revealed that Si.EtAc is more potent in relaxing K^+^-induced contractions, suggesting that Ca^+^^2^ channel blocking components are concentrated in organic fraction, which is in agreement with previous studies (Gilani et al. 2008; Janbaz et al. 2014). The potency of Si.EtAc in relaxing carbachol and K^+^-induced contractions was found greater than that of Si.Aq and Si.Cr, which suggests that the active components are concentrated in organic fraction.

Combination of Ca^+^^2^ antagonist and β-adrenergic agonist activities could have synergism in making the plant more effective against spasmodic conditions of gastrointestinal and respiratory systems (Gilani & Rehman 2005).

## Conclusions

The gut and tracheal relaxant activities of *Salsola imbricata* are mediated possibly through Ca^+^^2^ antagonist and β-adrenergic receptor agonist effects that explain its therapeutic usefulness in hyperactive gut and respiratory disorders, such as abdominal colic, diarrhoea, cough and asthma.
